# Preparation, Identification, Molecular Docking Study and Protective Function on HUVECs of Novel ACE Inhibitory Peptides from Protein Hydrolysate of Skipjack Tuna Muscle

**DOI:** 10.3390/md20030176

**Published:** 2022-02-27

**Authors:** Shuo-Lei Zheng, Qian-Bin Luo, Shi-Kun Suo, Yu-Qin Zhao, Chang-Feng Chi, Bin Wang

**Affiliations:** 1 Zhejiang Provincial Engineering Technology Research Center of Marine Biomedical Products, School of Food and Pharmacy, Zhejiang Ocean University, Zhoushan 316022, China; 15757488926@139.com (S.-L.Z.); 13275896859@163.com (S.-K.S.); zhaoy@hotmail.com (Y.-Q.Z.); 2 National and Provincial Joint Laboratory of Exploration and Utilization of Marine Aquatic Genetic Resources, National Engineering Research Center of Marine Facilities Aquaculture, School of Marine Science and Technology, Zhejiang Ocean University, Zhoushan 316022, China; iloveiceice@sina.cn

**Keywords:** skipjack tuna (*Katsuwonus pelamis*) muscle, angiotensin-I-converting enzyme (ACE) peptide, molecular docking, nitric oxide (NO), endothelin-1 (ET-1)

## Abstract

To prepare bioactive peptides with high angiotensin-I-converting enzyme (ACE)-inhibitory (ACEi) activity, Alcalase was selected from five kinds of protease for hydrolyzing Skipjack tuna (*Katsuwonus pelamis*) muscle, and its best hydrolysis conditions were optimized using single factor and response surface experiments. Then, the high ACEi protein hydrolysate (TMPH) of skipjack tuna muscle was prepared using Alcalase under the optimum conditions of enzyme dose 2.3%, enzymolysis temperature 56.2 °C, and pH 9.4, and its ACEi activity reached 72.71% at 1.0 mg/mL. Subsequently, six novel ACEi peptides were prepared from TMPH using ultrafiltration and chromatography methods and were identified as Ser-Pro (SP), Val-Asp-Arg-Tyr-Phe (VDRYF), Val-His-Gly-Val-Val (VHGVV), Tyr-Glu (YE), Phe-Glu-Met (FEM), and Phe-Trp-Arg-Val (FWRV), with molecular weights of 202.3, 698.9, 509.7, 310.4, 425.6, and 606.8 Da, respectively. SP and VDRYF displayed noticeable ACEi activity, with IC_50_ values of 0.06 ± 0.01 and 0.28 ± 0.03 mg/mL, respectively. Molecular docking analysis illustrated that the high ACEi activity of SP and VDRYF was attributed to effective interaction with the active sites/pockets of ACE by hydrogen bonding, electrostatic force, and hydrophobic interaction. Furthermore, SP and VDRYF could significantly up-regulate nitric oxide (NO) production and down-regulate endothelin-1 (ET-1) secretion in HUVECs after 24 h treatment, but also abolish the negative effect of 0.5 μM norepinephrine (NE) on the generation of NO and ET-1. Therefore, ACEi peptides derived from skipjack tuna (*K. pelamis*) muscle, especially SP and VDRYF, are beneficial components for functional food against hypertension and cardiovascular diseases.

## 1. Introduction

The incidence rates of cardiovascular diseases (CVDs), including coronary artery disease, rheumatic heart disease, hypertension and stroke, have increased noticeably over the past few years and have triggered the deaths of nearly 17.9 million people [1]. These chronic diseases are interrelated and associated with atherosclerosis, which is the primary cause of CVDs [2]. Endothelial dysfunction is recognized as a vital early event for the atherosclerosis developing process, preceding gross morphological-related signs and clinical-related symptoms [3]. Physiologically, endothelial cells are located inside intima, i.e., the vasculature’s inner lining and critically regulate vascular tone through the release of mediators (e.g., endothelin (ET-1) and nitric oxide (NO)) [4]. Angiotensin-I-converting enzyme (ACE) can convert angiotensin I (Ang I) to Ang II for inactivating the vasodilator bradykinin, and ACE inhibitory (ACEi) activity is a vital method for mediating systemic hypertension [5,6]. After captopril (Cap), as the first ACE inhibitor, was recommended by the FDA in 1981, several synthesized ACE inhibitors (e.g., enalapril and lisinopril), developed based on a snake venom peptide scaffold, have been used as anti-hypertensive agents in various clinical conditions and have shown some serious side effects [7]. Therefore, searching for functional peptide molecules for controlling endothelial dysfunction and inhibiting ACE is promising for atherosclerosis therapy and decreasing CVDs’ risk.

Currently, bioactive peptides have been prepared from a variety of protein resources and exhibit a variety of biological activities [8,9,10]. Among these peptides, ACEi peptides represent a promising alternative to synthetic drugs to control hypertension [11,12]. For instance, canary seed peptides (CSP) have great potential as anti-hypertensive and anti-obesity agents due to their inhibition activity on ACE and pancreatic lipase, and their non-competitive inhibitory activity was due to their destabilization of the transition state and Zn(II) coordination in ACE [13]. Noncompetitive ACEi peptides of TYLPVH (IC_50_ 1.37 μM) and GRVSNCAA (IC_50_ 57.93 μM) from *Ruditapes philippinarum* can lower systolic blood pressure in the single blood pressure test and promote NO secretion and reduced ET-1 production [14]. The ACEi peptide of KYIPIQ found in yak milk casein can promote NO synthesis and the expression of phosphorylated endothelial NO synthase (eNOS) in HUVECs by activating the protein kinase B (Akt) pathway [15]. Therefore, these results suggest that ACEi peptides derived from food protein resources have the potential to be good for human health on anti-hypertension.

Recently, many commercialization efforts have been made to produce functional food enriched with ACEi peptides. For instance, the fermentation technology of milk and soybean has been optimized to enhance the release of ACE inhibitory peptides, and the fermented products were applied to generate a type of antihypertensive function food [16]. Moreover, fermented antihypertensive milk products are available in the market, such as the Japanese brand Ameal S and Calpis (Calpis Co., Tokyo, Japan) containing IPP and VPP [17]. In addition, some lactic acid bacteria for producing ACEi peptides have been patented. For instance, *Lactobacillus helveticus* Cardi-04 (Chr. Hansen, Horsholm, Denmark) has been patented to prepare antihypertensive peptides and functional foods (WO 2003/082019 A3 and WO 2004/015125 A1). Additionally, a US patent (US 8,865,155) reported the production method of fermented milk with antihypertensive peptides using *L. lactis* (NRRL-B50571) [18]. However, limited efforts have been made to investigate the activity changes of ACEi peptides during different food processing, and further studies are required to optimize the manufacturing procedure of ACEi peptides in functional foods. 

Skipjack tuna (*Katsuwonus pelamis*) is a commercially important marine product worldwide and is considered a high-protein, low-fat, and low-calorie food [19,20]. Functional molecules such as collagens/gelatin, unsaturated fatty acid, protease, and polysaccharides have been generated from skipjack tuna and its by-products and exhibit potential application prospects and various biological activities, e.g., preventing arteriosclerosis, anti-inflammatory, and anticancer, as well as lowering blood cholesterol levels [21,22]. Presently, various bioactive peptides have been found in tuna by-products, such as bone/frame [23,24], scale [25], roe [20,26], and head and viscera [27,28]. In addition, Maeda et al., reported that the dietary dark muscle protein of tuna could reduce hepatic steatosis and promote serum high-density lipoprotein cholesterol in obese Type-2 diabetic/obese KK-A (y) mice [29]. Chi et al., found that protein hydrolysate of tuna muscle showed significant antioxidant activity [30]. These results indicate that tuna muscles are high-quality protein resources for the preparation of active peptides [30]. Therefore, the objectives of the research were to optimize the preparation process of ACEi protein hydrolysate of skipjack tuna muscle, identify the prepared ACEi peptides, and evaluate the bioactivity of the prepared ACEi peptides.

## 2. Results and Discussion

### 2.1. Amino Acid Composition of Skipjack Tuna Muscle

As presented in Table 1, skipjack tuna muscles were rich in Asp, Glu, Leu, and Lys and exhibited a 41.62% weight of essential amino acids/weight of amino acids (WEAA/WTAA) and 85.99% essential amino acids/weight of non-essential amino acids (WEAA/WNEAA). After defatting, the ratios of WEAA/WTAA and WEAA/WNEAA changed to 40.94% and 83.59%, respectively. FAO/WHO suggested that amino acids composed of high-quality protein exhibited nearly 40% WEAA/WTAA, as well as over 60% WEAA/WNEAA. The presented data indicate that the ratio of WEAA/WTAA of skipjack tuna muscle was slightly higher than 40%, and the ratio of WEAA/WNEAA was significantly greater than 60%. Therefore, skipjack tuna muscle can serve as a high-quality protein resource.

### 2.2. Preparation of Protein Hydrolysate of Skipjack Tuna Muscle

#### 2.2.1. Screening of Protease Species

**Figure 1:**
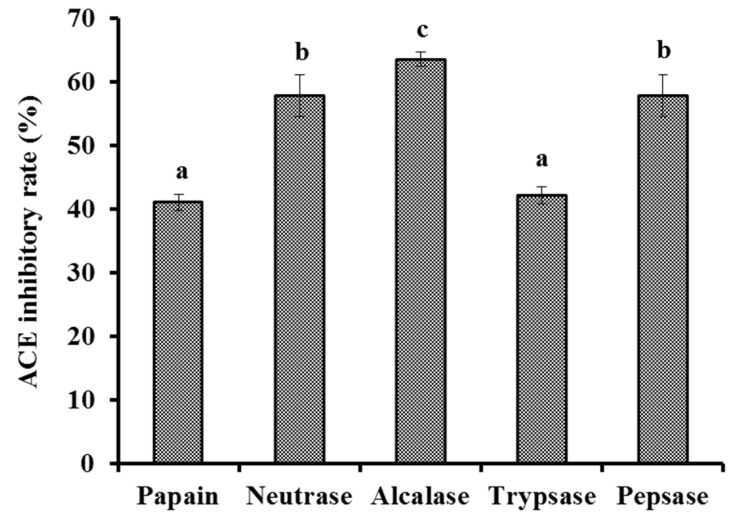
Effects of papain (55 °C, pH 7.0), pepsase (37.5 °C, pH 2.0), Alcalase (55 °C, pH 9.5), Neutrase (55 °C, pH 7.0), and trypsase (37.5 °C, pH 7.8) with enzyme dose of 2% (*w*/*w*) for 3h on the ACE inhibitory (ACEi) activity of protein hydrolysates from skipjack tuna muscle. ^a–c^ Values with same letters indicate no significant difference (*p* > 0.05).

Protein hydrolysates of skipjack tuna muscle were produced by five kinds of proteases (Figure 1). At 1.0 mg/mL, the ACEi rate of protein hydrolysate produced using Alcalase was 63.57 ± 1.13%, which was significantly higher than the rates of protein hydrolysates produced using papain (41.08 ± 1.25%), Neutrase (57.83 ± 3.23%), trypsase (42.19 ± 1.35%), and pepsase (57.83 ± 3.23%), respectively (*p* < 0.05). These biological functions of protein hydrolysates are in close contact with the structure of bio-peptides [12,31]. Thus, Alcalase, Neutrase, pepsase, papain, trypsase, and their combinations are frequently applied to yield protein hydrolysates from various protein sources [32,33,34]. The present results further supported the conclusion that the specificity of proteases could markedly influence the peptide composition and biological functions of protein hydrolysates [33,35]. Therefore, Alcalase was selected to prepare a protein hydrolysate of tuna muscle.

#### 2.2.2. Optimization of the Hydrolysis Conditions of Alcalase Using Single Factor Experiment

**Figure 2:**
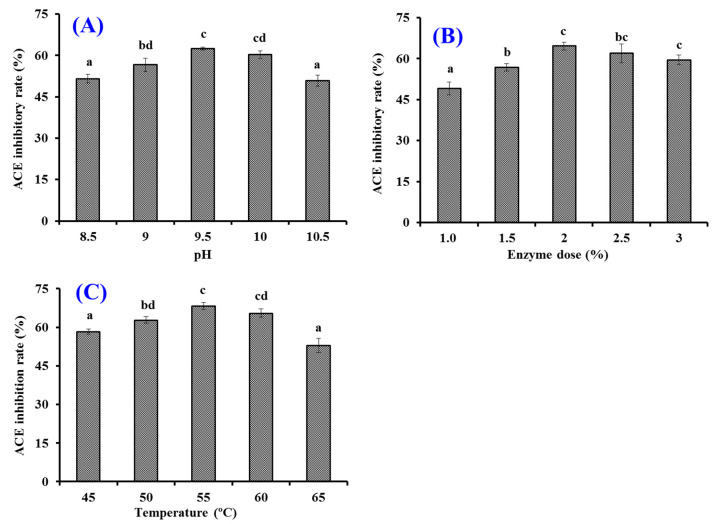
Effects of different hydrolysis conditions on ACEi activity of protein hydrolysates from skipjack tuna muscle. (**A**) pH; (**B**) enzyme dose (%); (**C**) temperature (°C). ^a–d^ Values with same letters indicate no significant difference (*p* > 0.05).

As shown in Figure 2, the effects of hydrolysis conditions of Alcalase including pH, temperature, and enzyme dose on the ACEi activity of protein hydrolysates were optimized by a single factor experiment. Figure 2A indicates that pH values significantly influenced the ACEi activity of protein hydrolysates, and the ACEi activity (56.57 ± 2.42% to 60.29 ± 1.44%) of protein hydrolysates prepared at pH 9 and 10 was significantly higher than those of protein hydrolysates prepared at pH 8.5 and 10.5 (*p* < 0.05). Figure 2B shows that the ACEi activity of protein hydrolysates was dramatically affected by the enzyme dose, and the ACEi activity (64.64 ± 1.37%) of protein hydrolysate prepared at the dose of 2.0% was significantly higher than those of hydrolysates prepared at other tested enzyme doses (*p* < 0.05). Additionally, the ACEi activity of protein hydrolysate slowly descended when the dose was higher than 2.0%. Figure 2C illustrates that the ACEi activity of hydrolysates increased remarkably when the temperature increased from 45 to 55 °C and achieved the highest value (68.33 ± 1.36%) at 55 °C, and there was a very significant decline in ACEi activity when the temperature was higher than 60 °C. Therefore, the range of hydrolysis conditions for Alcalase was narrowed to 9–10, 50–60 °C, and 1.5–2.5% for pH, temperature, and enzyme dose, respectively.

#### 2.2.3. Optimization of the Hydrolysis Conditions of Alcalase by Response Surface Experiment

According to the single-factor experiment data (Figure 2), the range of hydrolysis conditions for Alcalase was narrowed to 9–10, 50–60 °C, and 1.5–2.5% for pH, temperature, and enzyme dose, respectively. Moreover, ACEi activity under a response surface experiment design of three-level, three-factor factorial is presented in Table 2. After regression fitting of response values and variables in Table 2 by design-Expert 8.0.6, the quadratic multinomial regression equation disclosing the relationship between the ACEi rate (*Y*) and the variables (pH (*X*_1_), temperature (*X*_2_) and enzyme dose (*X*_3_)) was as given below:

*Y* (%) = 69.05 − 3.16X_1_ + 2.6*X*_2_ + 3.12*X*_3_ + 0.34*X_1_X_2_* + 3.63*X_1_X_3_* − 2.5*X_2_X_3_* − 4*X*_1_^2^ − 2.95*X*_2_^2^ − 1.89*X*_3_^2^ (1)

The results of the significance test of the coefficients of the regression model and variance analysis results of the equation are shown in Table 3, where *X*_1_, *X*_2_, *X*_3_, *X_1_X_3_*, *X_2_X_3_*, *X*_1_^2^, and *X*_2_^2^ had significant negative effects on ACEi rate with *p* < 0.05. The results showed that the effects of various variances on enzymatic hydrolysis process parameters had an interaction effect, rather than a simple linear relationship. The determination coefficient (*R^2^*) of ACEi rate was 0.9689, meaning that 96.89% of the observed results could be fitted well by this regression equation. The relationship between independent variables and the response value could be well described by the regression equation. Therefore, the equation can reliably be applied to predict the optimal process conditions for preparing ACEi hydrolysate of skipjack tuna muscle by Alcalase. In addition, the analysis results in Table 3 indicated that the order of influence of each variance on ACEi rate was: enzyme dose (*X*_3_) > pH (*X*_1_) > temperature (*X*_2_).

**Figure 3:**
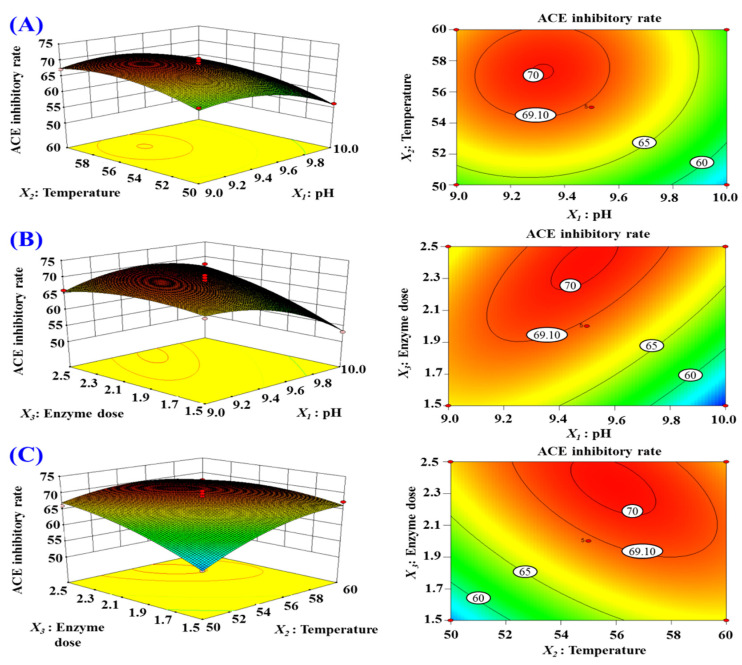
Response surface graph for ACEi rate as a function of (**A**) temperature and pH, (**B**) enzyme dose and pH, and (**C**) enzyme dose and temperature during the hydrolysis of skipjack tuna muscle with Alcalase.

According to the regression equation, the 3D response surface results of multiple non-linear regression models (Figure 3) were applied to illustrate the interactive influence of the variables and their mutual interaction on the ACEi rate. As shown in Figure 3A, ACEi rate increases to a certain extent when pH value increases within a certain range, but ACEi rate decreased instead of increasing when the pH value exceeded this range. The effect of temperature (Figure 3B) and enzyme dosage (Figure 3C) showed the similar trends. The shape (elliptic or round) of contour map can reflect the strength and significance of the interaction between two independent variables. Elliptic indicates that the interaction between the two independent variables is evident; round indicates that the interaction between the two factors is not evident. The contour maps of Figure 3B,C were elliptic, indicating that the influence between the two factors (*X_1_X_3_* and *X_2_X_3_*,) was evident. The contour map of Figure 3A tended to be round, indicating that the interaction between pH and temperature was not significant when other factors were fixed. These results agreed well with the data summarized in Table 2. According to the analysis of Design-Expert 8.0.6 software, the optimal processing conditions of Alcalase for preparing protein hydrolysate of tuna muscle were as follows: pH 9.4, enzyme dosage 2.3%, and temperature 56.2 °C. Using optimum hydrolysis conditions, the ACEi rate of prepared hydrolysate (referred to as TMPH) of tuna muscle was 72.71%, which was very close to the predicted 73.20% and confirmed the validity and adequacy of the predicted equation.

### 2.3. ACEi Peptides Prepared from TMPH

#### 2.3.1. Ultrafiltration of TMPH

**Figure 4:**
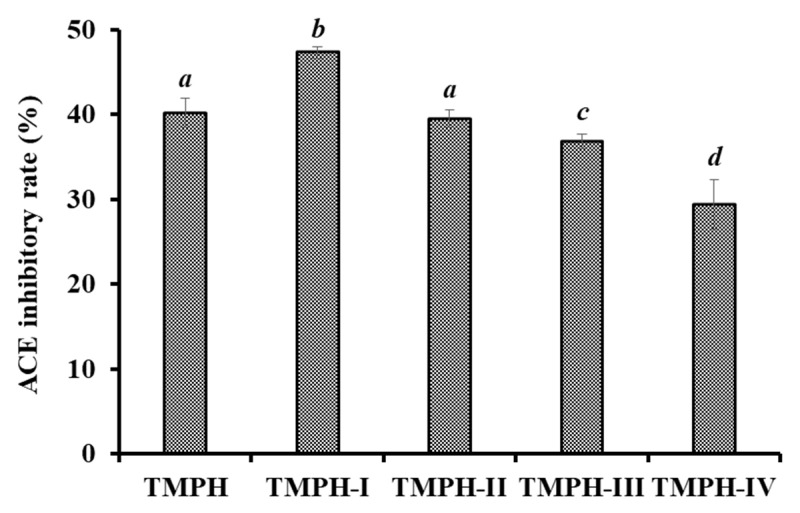
ACEi rates of ultrafiltration fractions (TMPH-I, TMPH-II, TMPH-III and TMPH-IV) of TMPH at 0.5 mg/mL. ^a–d^ Values with same letters indicate no significant difference (*p* > 0.05).

Through 3.5, 5, and 10 kDa ultrafiltration membranes, the TMPH underwent the fractionation into four different MW fractions (TMPH-I, TMPH-II, TMPH-III, and TMPH-IV). According to Figure 4, the ACEi activity of TMPH-I was 47.26 ± 0.64% at 0.5 mg/mL (*p* < 0.05), which was remarkably higher than those of TMPH (40.17 ± 1.74%), TMPH-II (39.48 ± 1.05%), TMPH-III (36.84 ± 0.86%), and TMPH-IV (29.38 ± 2.88%), respectively. Polypeptides with large size are difficult to enter and bind to the active site of ACE, resulting in a decrease in their inhibitory activity [12]. Therefore, ultrafiltration technology is often applied to pool bioactive peptides with small MWs from protein hydrolysates [36,37,38]. The present data were in agreement with the reported literatures that low MW peptide fractions of protein hydrolysates from *Saurida elongate* [39], tuna frame [24], Antarctic krill [6], *Katsuwana pelamis* [40], *Okamejei kenojei* [41], and squid (*Dosidicus gigas*) skin [42] had the highest ACEi activity. Then, TMPH-I with the smallest MW revealed strong ACEi activity and was selected for further separation.

#### 2.3.2. Gel Permeation Chromatography (GPC) of TMPH-I

**Figure 5:**
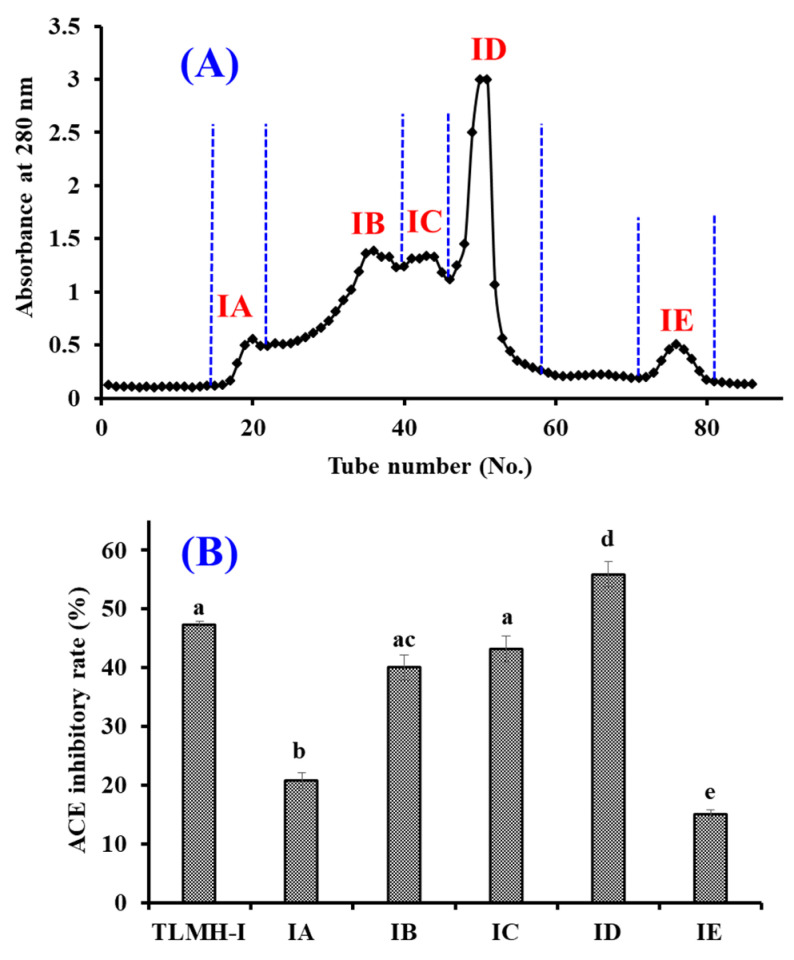
Chromatogram profile of TMPH-I isolated by Sephadex G-25 (**A**) and the ACEi rates of prepared subfractions (IA-IE) from TMPH-I at 0.5 mg/mL (**B**). ^a–e^ Values with same letters indicate no significant difference (*p* > 0.05).

TMPH-I was divided into five peptide fractions (IA-ID) by Sephadex G-25 chromatography and their ACEi rates were shown in Figure 5. At 0.5 mg/mL, the ACEi rate of ID was 55.87 ± 2.15%, which was remarkably (*p* < 0.05) higher than those of TMPH-I (47.26 ± 0.64%), IA (20.77 ± 1.36%), IB (39.99 ± 2.13%), IC (43.19 ± 2.19%), and IE (15.07 ± 0.72%), respectively (Figure 5). Then, the peptide fraction of ID was chosen for RP-HPLC isolation.

#### 2.3.3. RP-HPLC Purification of TMPH-ID

**Figure 6:**
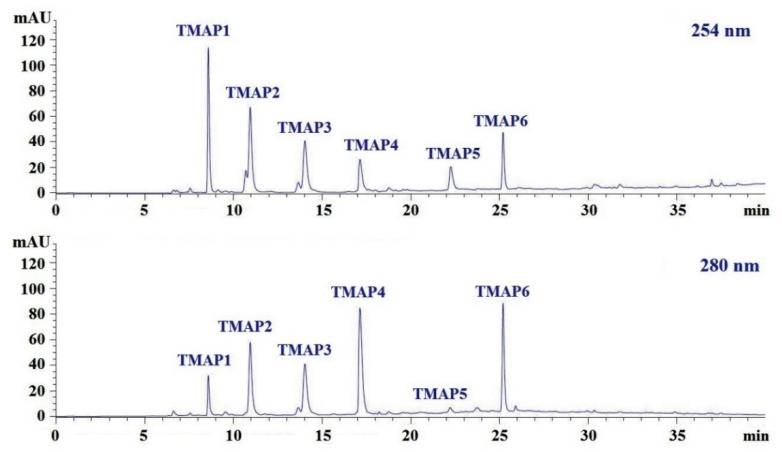
Elution profiles of subfraction ID by RP-HPLC using a gradient of acetonitrile containing 0.06% trifluoroacetic acid at 254 nm and 280 nm.

ID was finally purified by RP-HPLC with a Zorbax 300SB-C18 column (9.4 × 250 mm, 5 μm) (Figure 6). According to the elution profiles of ID fraction at 254 and 280 nm, six peptides with retention times of 8.590 min (TMAP1), 10.950 min (TMAP2), 14.032 min (TMAP3), 17.139 min (TMAP4), 22.258 min (TMAP5), and 25.209 min (TMAP6) were collected and lyophilized (Table 4).

### 2.4. Peptide Sequences and MWs Determination

**Figure 7:**
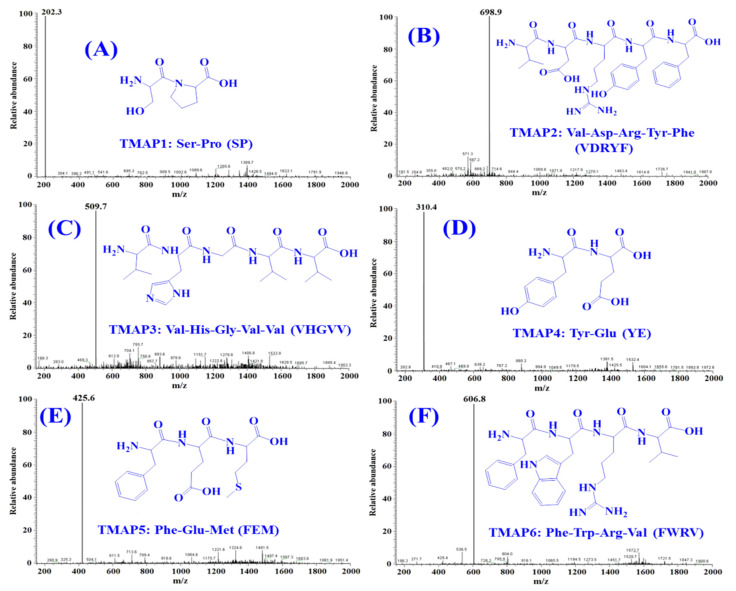
Mass spectrogram of six ACEi peptides (TMAP1-TMAP6) from protein hydrolysate of skipjack tuna muscle (STPM). (**A**) TMAP1; (**B**) TMAP2; (**C**) TMAP3; (**D**) TMAP4; (**E**) TMAP5; (**F**) TMAP6.

By employing Protein/Peptide Sequencer, six peptides (TMAP1-TMAP6) sequences were identified as Ser-Pro (SP, TMAP1), Val-Asp-Arg-Tyr-Phe (VDRYF, TMAP2), Val-His-Gly-Val-Val (VHGVV, TMAP3), Tyr-Glu (YE, TMAP4), Phe-Glu-Met (FEM, TMAP5), and Phe-Trp-Arg-Val (FWRV, TMAP6), respectively, and their MWs were determined as 202.3, 698.9, 509.7, 310.4, 425.6, and 606.8 Da, respectively (Figure 7), which were in good agreement with their theoretical MWs (Table 4).

### 2.5. Bioactive Properties of Six ACEi Peptides (TMAP1-TMAP6)

#### 2.5.1. ACEi Activity and Molecular Docking Analysis

Table 4 showed that the IC_50_ values of TMAP1 and TMAP2 on ACE were 0.06 ± 0.01 and 0.28 ± 0.03 mg/mL, which were significantly lower than those of TMAP3 (0.90 ± 0.16 mg/mL), TMAP4 (0.80 ± 0.03 mg/mL), TMAP5 (2.18 ± 0.20 mg/mL), and TMAP6 (0.76 ± 0.10 mg/mL), respectively (*p <* 0.05). Additionally, the IC_50_ vales of TMAP1 and TMAP2 were lower than those of ACEi peptides from protein hydrolysates of seaweed pipefish (HWTTQR: 1.44 mg/mL) [43], Antarctic krill (WF: 0.32 mg/mL; YAK: 1.26 mg/mL; FQK: 1.76 mg/mL) [6]), skate (MVGSAPGVL: 3.09 mg/mL; LGPLGHQ: 4.22 mg/mL) [44], stone fish (LAPPTM: 1.31 mg/mL; EVLIQ: 1.44 mg/mL; EHPVL: 1.68 mg/mL) [45], and lizardfish (AGPPGSDGQPGA: 544.10 mg/mL) [46], respectively. The present results demonstrate that TMAP1-TMAP6, especially TMAP1 and TMAP2, had significantly ACEi activity and could serve as functional molecules applied in antihypertensive products.

**Figure 8:**
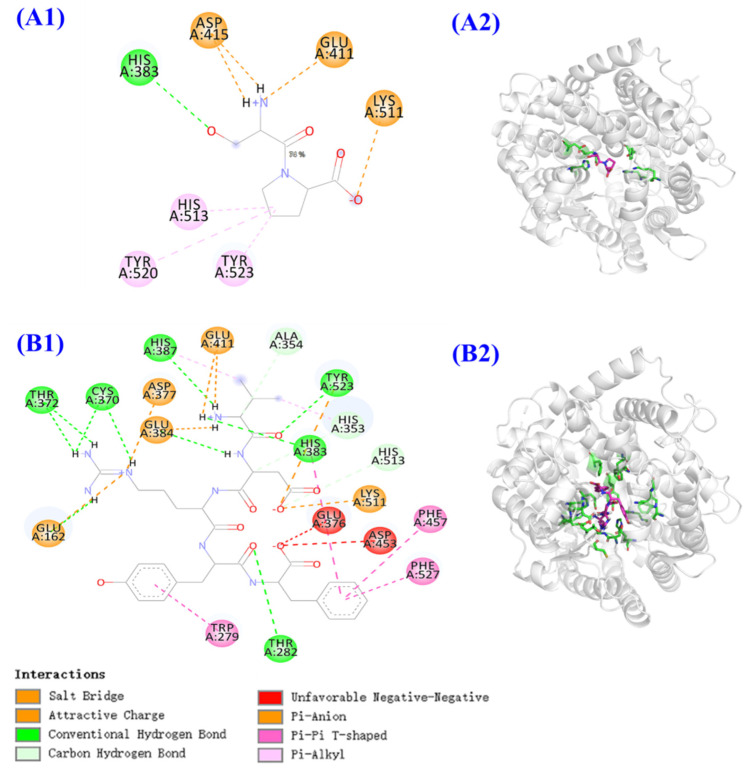
Molecular docking results of TMAP1 and TMAP2 with ACE. (**A1**) 2D details of ACE and TMAP1 interaction. (**A2**) 3D interaction details for TMAP1. (**B1**) 2D details of ACE and TMAP2 interaction. (**B2**) 3D interaction details for TMAP2.

A molecular docking experiment was performed to analyze the mechanism of ACEi peptides (TMAP1 and TMAP2) and ACE (Figure 8). ACE has three major active site pockets (S1, S2, and S1’). The S1 pocket includes Ala354, Glu384, and Tyr523 residues; the S2 pocket includes Gln281, His353, Lys511, His513, and Tyr520 residues; and S1’ contains Glu162 residues [12]. Cap is a widely recognized ACE inhibitor and interacts at the sites of Gln281, His353, Ala354, Glu384, Lys511, His513, Tyr520, and Tyr523 residues of ACE, which indicates that these amino acid residues play key roles in ACE binding [12]. Figure 8A indicated that TMAP1 formed a hydrogen bond with His383 residue and a hydrophobic interaction with His513, Tyr523, and Tyr520 residues of ACE, and interacted with Asp415, Glu411, and Lys511 residues of ACE by electrostatic force. Figure 8B proved that TMAP2 (VDRYF) formed hydrogen bonds with Ala354, His353, His513, Tyr523, His383, Thr282, His387, Cys370, and Tyr372 residues of ACE, among which it formed hydrogen bonds with active pockets of S1 (Ala354 residue) and S2 (His353 and His513 residues). In addition, TMAP2 (VDRYF) interacted with Phe457, Phe527, and Trp279 residues of ACE through hydrophobic effect and interacted with Glu411, Asp377, Glu384, Glu162, and Lys511 residues of ACE through electrostatic force. The molecular docking analysis demonstrated that TMAP1 and TMAP2 exhibit better ACEi activity, attributed to the effective interaction with the active sites of ACE by hydrogen bonding, electrostatic force, and hydrophobic interaction. Meanwhile, the ACEi activity of TMAP2 should be related to the interaction with active pockets.

The molecular docking analysis indicated that the affinity of TMAP1 and TMAP2 with ACE was −5.7 and −9.7 kcal/mol, which was similar to those of YSK (−7.9 kcal/mol) [47], YVVF (−9.8 and −8.8 kcal/mol for C-domain and N-domain affinity), WMY (−9.3 and −8.5 kcal/mol for C-domain and N-domain affinity), and LVLL (−8.6 and −7.5 kcal/mol for C-domain and N-domain affinity) [48].

Molecular size and amino acid sequence are two critical factors influencing the ACEi ability of antihypertensive peptides [49,50]. Molecular size determines the affinity of a peptide with the ACE active site because large peptides cannot accommodate to the narrow binding channel of ACE [12]. Abdelhedi et al. reported that ACEi tripeptides VPP and IPP could easily enter the ACE channel and coordinate with Zn^2+^, but larger peptides composed of 7–11 amino acid residues, such as ALPMHIR, VKPLPQSG, AVVPPSDKM, GPAGPRGPAG, and TTMYPGIA, showed low interaction scores combining with the ACE active site [51]. In the study, TMAP1 and TMAP2 were dipeptides or pentapeptides, and the small size increased their access to the binding channel of the ACE active site.

The amino acid sequence is another factor affecting the ACEi ability of antihypertensive peptides. ACE consists of C- and N-domains, and each contains a binding active site of zinc cofactor [52]. Therefore, the C- and N-terminal amino acids are important to the activity of antihypertensive peptides. Presently, the kind of C-terminal amino acids on the ACEi activity and mechanism has been widely discussed. Zheng et al. indicated that branched-chain (Leu and Ile) and aromatic (Tyr and Phe) amino acids or Pro were the main residues in their C-terminal tripeptides [53]. Hayes et al. found that hydrophobic amino acids, such as Phe, Trp, Tyr, Val, Leu, and Ile, favored combination with the C-terminal active site of ACE [54]. Pro has aroused great attention amongst researchers because Pro occurs frequently at the C-terminus of antihypertensive peptides, such as LYPPP, YSMYPP, VGLYP, EVSQGRP, YP, and KDEDTEEVP, and could enhance the resistant ability of peptides against gastro-intestinal digestion and kept good bioavailability [4]. Therefore, Pro and Phe residues at the C-terminus of TMAP1 and TMAP2 are particularly critical for their ACEi activity.

The function of N-terminal amino acids is less discussed compared with C-terminal amino acids. Auwal et al. illustrated that branched aliphatic or dicarboxylic amino acids (Val, Ala, Ile, and Leu) at the N-terminus could strengthen ACEi ability of peptides [45]. Then, Val residue at the N-terminus of TMAP2 could improve its ACEi ability. In addition, Ser residue was found in some ACEi peptides, such as VSQLTR (IC_50_ 105 μM) [55], YSK (IC_50_76 mM) [47], and SPRCR (IC_50_ 41 μM) [56]. Therefore, Ser residue, especially its hydroxyl group, should play a vital effect in the ACEi capability of TMAP1, and the result was confirmed by Figure 8B that the hydroxyl group of Ser residue formed hydrogen bonds with the His383 residue of ACE.

#### 2.5.2. Effects of TMAP1 and TMAP2 on HUVECs

##### Effects of TMAP1 and TMAP2 on Cell Viability

**Figure 9:**
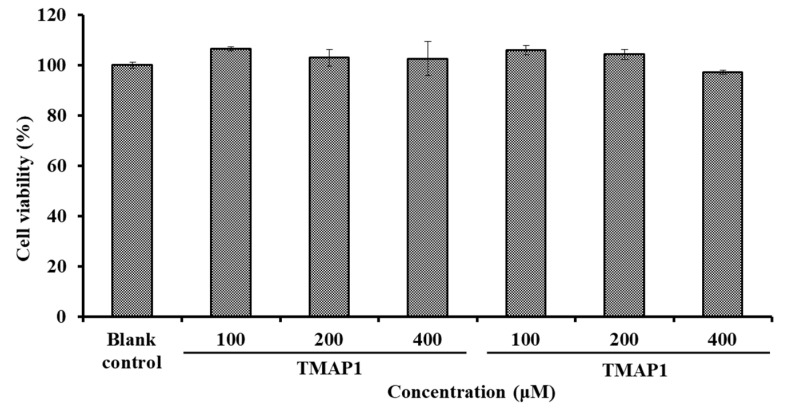
The cell viability of HUVECs treated with TMAP1 and TMAP2 for 24 h.

The effects of TMAP1 and TMAP6 on the viability of HUVECs at concentrations of 100–400 μM were shown in Figure 9. After being incubated for 24 h at the determined concentrations, the cell viability of the TMAP1 group ranged from 102.63 ± 6.79% to 106.64 ± 0.82%, and the cell viability of the TMAP2 group ranged from 97.18 ± 0.86% to 106.05 ± 1.91%. The cell viability of the TMAP1 and TMAP2 groups was higher than 90% of the blank control, which indicated that TMAP1 and TMAP2 did not induce significant cell toxicity in HUVECs.

Vascular endothelial cells enshroud the inner surface of blood vessels and are crucial regulatory factors of vascular tone through generating vasodilatory and vasoconstrictory agents. Thus, HUVECs are usually used as model cells for illustrating the mechanism of blood pressure regulation [4,12]. Cells maintain an appropriate balance between proliferation and death in normal tissues, and bioactive compounds with strong cell proliferation inhibition indicate their potential toxicity to normal organs and tissues, and are thought to be ill-suited to the development of non-antitumor health products [12,57]. The present results indicate that TMAP1 and TMAP2 were not significantly toxic to HUVECs and should be suitable for producing antihypertensive products.

##### Effects of TMAP1 and TMAP2 on NO Production

**Figure 10:**
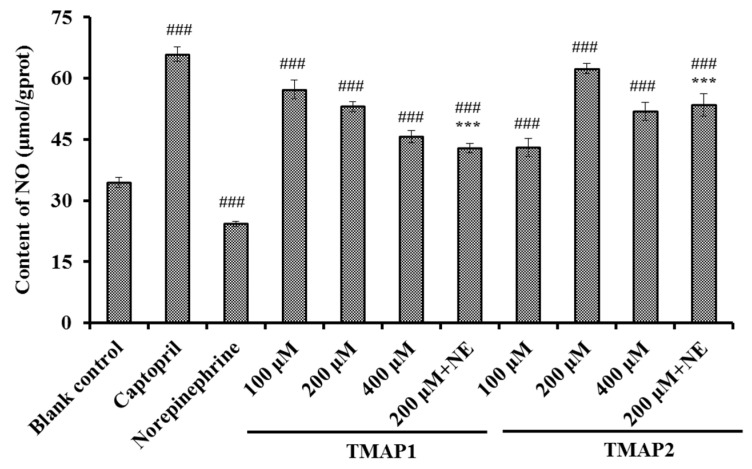
The production of nitrico xide (NO) of HUVECs treated with TMAP1 and TMAP2 for 24 h. Cell group treated with captopril (Cap) was designed as a positive control. ^#^^##^ *p* < 0.001 vs. control group; *** *p* < 0.001 vs. norepinephrine (NE) group.

According to Figure 10, Cap could significantly increase the level (65.96 ± 1.83 μmol/gprot) of NO in HUVECs in comparison with the control group (34.41 ± 1.27 μmol/gprot) (*p* < 0.001). Similarly, the NO levels in HUVECs treated with TMAP1 and TMAP2 at 100, 200, and 400 μM were significantly increased compared with the control group (*p* < 0.001), and the NO levels of TMAP1 and TMAP2 groups increased to 57.25 ± 2.31 and 51.88 ± 2.16 μmol/gprot at 400 μM. Additionally, NE could significantly decrease the level of NO (24.25 ± 0.63 μmol/gprot) compared with the control group (*p* < 0.001), but the negatively influence of NE on reducing NO content was partially offset by TMAP1 and TMAP2 treatment at a 200 μM concentration (*p* < 0.001).

NO can antagonize the vascular tone function of angiotensin II by down-regulating the synthesis of ang II type 1 receptor and ACE, which is further involved in regulating the peripheral and central function of the cardiovascular system and exerts vasoprotective effects [4]. In addition, NO refers to the most potent vascular endothelium-derived vasodilator, but its formation is decreased in atherosclerosis [58]. Therefore, the deficiency of NO will increase the risks of cardiovascular in pathologic situations, and improving endothelial NO production represents reasonable therapeutic strategy for atherosclerosis [6]. Previous studies have shown that some ACEi peptides, such as WF, YRK, FQK, FAS, GRVSNCAA, and TYLPVH, exerted their antihypertensive activity by increasing the NO production of HUVEC [38]. The present data indicate that TMAP1 and TMAP2 could significantly increase the production of NO in HUVECs and even reverse the downtrend of NO production caused by NE.

##### Effects of TMAP1 and TMAP2 on ET-1 Secretion

**Figure 11:**
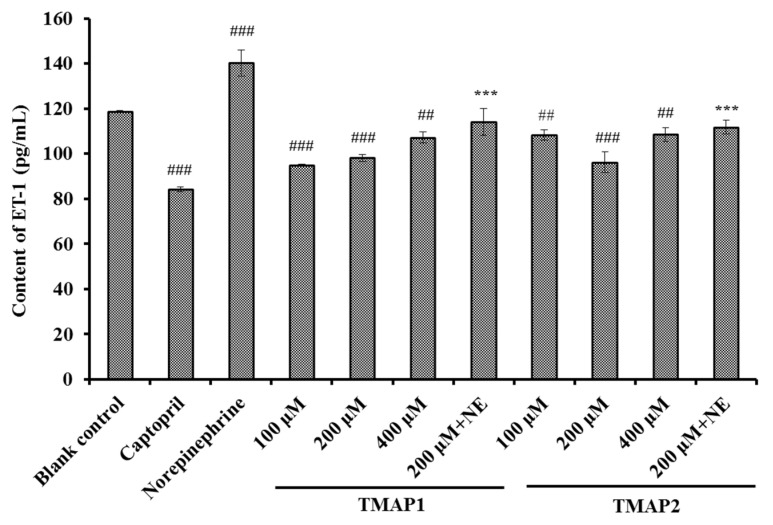
The endothelin-1 (ET-1) secretion of HUVECs treated with TMAP1 and TMAP2 for 24 h. Cell group treated with captopril (Cap) was designed as a positive control. ^#^^##^ *p* < 0.001 and ^##^ *p* < 0.01 vs. control group; *** *p* < 0.001 vs. norepinephrine (NE) group.

According to Figure 11, the ET-1 secretion of HUVECs was significantly decreased to 84.16 ± 1.18 pg/mL by 0.5 μM Cap treatment compared with the control group (118.68 ± 0.53 pg/mL) (*p* < 0.001). In addition, the ET-1 secretion of HUVECs significantly (*p* < 0.001 or *p* < 0.01) decreased by TMAP1 and TMAP2 under the tested concentrations, and the ET-1 levels of TMAP1 and TMAP2 groups reduced to 94.86 ± 0.39, and 92.09 ± 4.58 pg/mL at 400 μM. Conversely, NE could significantly increase ET-1 secretion (140.23 ± 5.81 pg/mL) compared with the control group (*p* < 0.01), but the ET-1 secretion negatively affected by NE was partially abrogated by TMAP1 and TMAP2 treatment, and ET-1 secretion decreased to 114.08 ± 5.82 pg/mL and 111.78 ± 3.06 pg/mL at 200 μM (*p* < 0.01).

ET-1 is a known vasoconstriction factor similar to Ang II and is capable of inducing endothelial dysfunction related to atherosclerosis and hypertension [6,59]. Zhang et al. reported that GRVSNCAA and TYLPVH from *R. philippinarum* played their anti-hypertension function through significantly lessening ET-1 generation [38], and ACEi peptides of WF, YRK, FQK, and FAS from Antarctic krill showed a similar activity of reducing ET-1 content [6]. The available results illustrated that TMAP1 and TMAP2 displayed similar capabilities to significantly decrease the secretion of ET-1 and reverse the uptrend of ET-1 secretion caused by NE in HUVECs.

As suggested from the mentioned outcomes, ACEi peptides from skipjack tuna muscle noticeably facilitate NO production while controlling ET-1 secretion in HUVECs. In addition, the peptides reversed the impact of NE upon NO- and ET-1-secreting processes in HUVECs. According to the mentioned findings, the ACEi peptides isolated from skipjack tuna muscle exert protection-related effects upon vascular endothelial functions and display an antihypertensive mechanism analogous to that of Cap.

## 3. Materials and Methods

### 3.1. Materials

Skipjack tuna (*K. pelamis*) muscle was provided by Ningbo Today Food Co., Ltd (China). Sephadex G-25 resins and NO assay kit (Nitrate reductase approach, A012-1) were purchased from Nanjing Jiancheng Bioengineering Institute (China). ET-1 ELISA Kit (HM10108) was purchased from Shanghai Qiaodu Biotechnology Co. Ltd. (China).Trypsase (≥20,000 U/g), pepsase (≥500,000 U/g), papain (≥400,000 U/g), ACE (A6778) and N-[3-(2-Furyl) acryloyl]-Phe-Gly-Gly (FAPGG, F713) were purchased from Sigma-Aldrich (Shanghai) Trading Co., Ltd. (China). Alcalase (B8360, 2.0 × 10^5^ U/g), 3-(4,5-Dimethylthiazol-2yl)-2,5-diphenyltetrazolium bromide (MTT; M8180), Penicillin-Streptomycin Liquid (P1400), dimethylsulfoxide (DMSO), fetal bovine serum (FBS; 11011-8611), HEPES buffer (H8090), norepinephrine (NE; IN0530), and Cap (C7510) were purchased from Beijing Solarbio Science & Technology Co., Ltd. (China). ACEi Peptides of (TMAP1-TMAP6) with purity higher than 98% were synthesized in Shanghai Apeptide Co. Ltd. (China).

### 3.2. Determination of Amino Acid Composition and ACEi Activity

Amino acid composition of skipjack tuna muscle was determined according to the previous method [60].

The ACEi activity was determined by employing FAPGG as the substrate with the previous protocol [6]. The assay was conducted in a Tris-HCl buffer (50 mM, pH 8.3) containing 300 mM NaCl. The same buffer was used to dilute the protein hydrolysates, enzyme, and substrate. The initial assay volume consisted of 50 µL of the substrate (3 mM), 50 µL of the ACE enzyme solution containing 1.25 mU of declared enzyme activity, and 50 µL of assay sample. All these solutions were incubated for 30 min at 37 °C in a water bath first without mixing and then for an additional 30 min after mixing. Glacial acetic acid (150 µL) was added to stop ACE activity. The reaction mixture was separated by HPLC to determine the hippuric acid (HA) content produced due to ACE activity on the substrate. The content of free HA was determined using a HPLC system (Agilent 1200, Agilent Ltd., Santa Clara, CA, USA) on a Zorbax, SB C-18 column (4.6 × 250 mm, 5 µm) using an isocratic system (pH 3.0) consisting of 12.5% (*v/v*) acetonitrile in deionised water. The sample (10 μL) was eluted at a flow rate of 1.0 mL/min and measured by monitoring the absorbance at 228 nm. In the HPLC method, the fitted linear equation between the peak area (y) and HA content (x) was calculated by the method of least squares, as y = 6052 x − 4.9429 (*R*^2^ = 0.9998). The HA content was calculated by the peak area. The control reaction mixture contained 50 µL of buffer instead of the assay sample, and the control was expected to liberate the maximum amount of HA from the substrate due to uninhibited ACE activity. The percent inhibition of ACE activity was calculated as follows:

Inhibition activity (%) = [(HA control − HA sample)/HA control] × 100%.

### 3.3. Preparation of Protein Hydrolysate of Skipjack Tuna Muscle

#### 3.3.1. Screening of Protease Species

The skipjack tuna muscle was pounded into a homogenate and defatted, as previously described [61]. The homogenate and isopropanol were mixed in a ratio of 1:4 (*w/v*) and stirred continuously for 4 h at 35 °C. Isopropanol was replaced every 2 h. The precipitate was collected by centrifugation at 9000 rpm for 15 min at 4 °C, freeze-dried, and stored at −20 °C.

Defatted tuna muscle powders were dispersed in distilled water (1%, *w/v*) and hydrolyzed for 3 h with an enzyme dose of 2% (*w/w*) using papain (55 °C, pH 7.0), pepsase (37.5 °C, pH 2.0), Alcalase (55 °C, pH 9.5), Neutrase (55 °C, pH 7.0), and trypsase (37.5 °C, pH 7.8), respectively. After that, the enzymolysis reaction was stopped at 95 °C for 20 min and centrifuged at 8000× *g* for a quarter of an hour at −4 °C. The resulting supernatant was lyophilized and stored at −20 °C. The protein hydrolysate produced by Alcalase showed the highest ACEi activity.

#### 3.3.2. Optimization of the Hydrolysis Conditions of Alcalase

A single-factor experiment was applied to optimize the hydrolysis conditions of Alcalase. pH (8.5, 9.0, 9.5, 10.0, and 10.5), hydrolysis temperature (45 °C, 50 °C, 55 °C, 60 °C, and 65 °C), and enzyme dose (1.0%, 1.5%, 2.0%, 2.5%, and 3.0%) were chosen for the present investigation.

According to the single-factor experiment results, a response surface methodology was employed to estimate the influence of independent variables (*X*_1_, pH; *X*_2_, temperature; *X*_3_, enzyme dose) in glycine-sodium hydroxide buffer (0.05 M) on ACEi activity [62,63]. The Box–Behnken design of three levels (*X*_1_: 9, 9.5, and 10; *X*_2_: 50, 55, and 60 °C; *X*_3_: 1.5, 2.0, and 2.5%) was used to analyze the effects of three variables on ACEi activity. The experimental operation after hydrolysis is the same as in Section 3.3.13.3.1. The protein hydrolysate prepared under the optimal enzymolysis conditions was referred to as TMPH.

### 3.4. Separation Process of ACEi Peptides from TMPH

**Figure 12:**
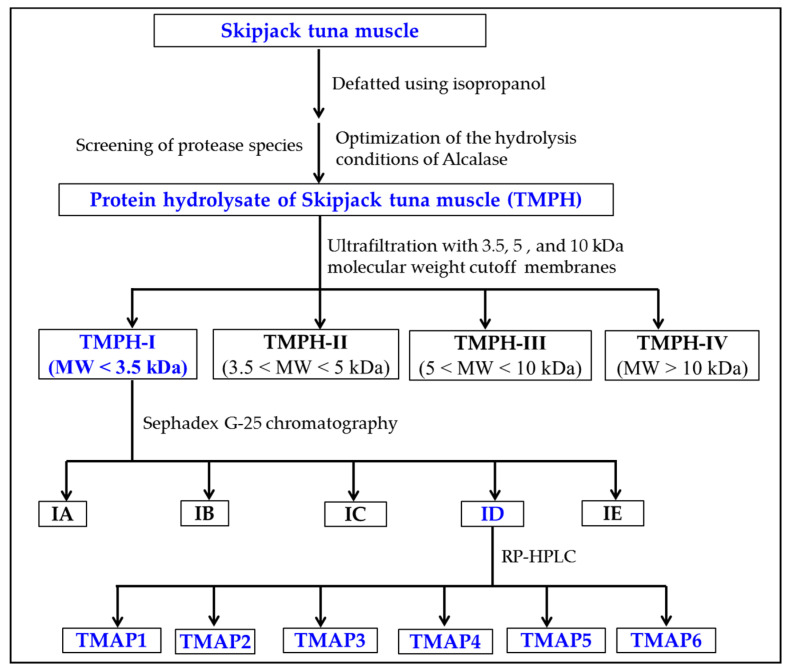
Flow diagram of purifying ACEi peptides from protein hydrolysate (TMPH) of skipjack tuna muscle prepared using Alcalase.

ACEi peptides were purified from TMPH using the following designed process (Figure 12).

TMPH (100.0 mg/mL) was fractionated with three MW cutoff membranes (10, 5, and 3.5 kDa) at 0.50 MPa, 4 °C, and four fractions including TMPH-I (<3.5 kDa), TMPH-II (3.5–5 kDa), TMPH-III (5–10 kDa), and TMPH-IV (>10 kDa) were collected and lyophilized. TMPH-I exhibited the maximum ACEi activity among four fractions.

TMPH-I solution (5 mL, 50.0 mg /mL) was fractionated with Sephadex G-25 column (3.6 × 150 cm) eluted with ultrapure water under 0.6 mL/min flow rate. Each eluate (1.8 mL) was collected by monitoring absorbance at 280 nm. Five subfractions (IA, IB, IC, ID, and IE) were isolated from TMPH-I solution and lyophilized. The ACEi activity of ID was higher than those of the other four fractions.

The ID solution (20 μL, 100.0 μg/mL) received final separation by RP-HPLC on a Zorbax 300SB-C18 column (9.4 × 250 mm, 5 μm) with a linear gradient of acetonitrile (1% acetonitrile in 7 min; 1–10% acetonitrile in 7 min; 10–30% acetonitrile in 7 min; 30–60% acetonitrile in 7 min; 60–100% acetonitrile in 7 min; and 100 B in 5 min) inside 0.06% trifluoro acetic acid (TFA) at 2.0 mL/min flow rate. The eluate absorbance was monitored at 254 and 280 nm. Six peptides (TMAP1 to TMAP6) were collected according to the elution chromatogram, dialyzed with MW cut-off dialysis tube of 100 Da, lyophilized, and followed by in-depth analysis for their amino acid sequences.

### 3.5. Identification of Sequence and MWs of ACEi Peptide

The sequences of TMAP1 to TMAP6 were analyzed using an Applied Biosystems 494 protein sequencer (Perkin Elmer, USA) [58]. Edman degradation was performed according to the standard program supplied by Applied Biosystems.

The precise MWs of TMAP1 to TMAP6 were determined by employing a Q-TOF mass spectrometric device (Micromass, Waters, Milford, MA, USA) in the combination of an electrospray ionization (ESI) source [64]. Nitrogen was maintained at 40 psi for nebulization and 9 L/min at 350 °C for evaporation temperature. The data were collected in centroid mode from *m/z* 200 to 2000.

### 3.6. Molecular Docking Experiment of TMAP1 and TMAP2

The molecular docking experiment was commissioned to Shanghai NovoPro Biotechnology Co., Ltd (Shanghai, China). The crystal structure of human ACE-lisinopril complex (1O8A.pdb) and Cap were acquired from the RCSB PDB Protein Data Bank (PDB code: 1UZF). The interaction between ACE and MCO was analyzed to determine the position and size of the binding pocket using Chimera software. All non-standard residues in the 1UZF model were deleted, and AutodockTools were used to convert PDB files into PDBQT files (adding Gasteiger charge and setting key distortion). Peptide molecules were converted into SMILES format by PepSMI tool, 3D models were drawn by Discovery Studio program, and energy minimization was performed using steepest-descent and conjugate-gradient techniques. Molecular docking and free energy calculation were carried out using flexible docking tool of Autodock Vina. Finally, the interaction between ACE and peptide molecules was analyzed by Chimera software. The best ranked docking poses of TMAP1 and TMAP2 in the active site of ACE were acquired on the binding-energy value and scores.

### 3.7. Effects of TMAP1 and TMAP2 on HUVECs

#### 3.7.1. HUVECs Culture and Cell Viability Assessment using MTT Assay

After thawing, HUVECs were maintained in cultured flasks and cultured to confluence in DMEM containing 1% Penicillin-Streptomycin liquid, supplemented with 10% FBS. HUVECs received the culturing process at 37°C in a humidified 5% CO_2_ atmosphere [6].

The cell viability of TMAP1 and TMAP2 on HUVECs were assessed using the MTT assay on manufacturer’s instructions [65,66]. In brief, cells were incubated in 96-well plates at density of 0.8 × 10^4^ cells per well with 180 μL completed DMEM. Following a confluency of 50–60% in the 96-well plates, the cells were treated with 20 μL peptides under designed concentrations (100, 200, and 400 μM) and further cultured for 24 h at 37 °C. Subsequently, the cells were added to 20 μL MTT solution (5 mg/mL) and incubated for 4 h. Finally, DMSO was added into each well, and the absorbance (A) at 490 nm was determined. The cell viability was calculated as:

Cell viability (%) = (A_experiment group_/A_control group_) × 100 (2)

#### 3.7.2. Evaluation of NO and ET-1 Production

The NO and ET-1 contents of HUVECs were determined after a 24 h treatment of ACEi peptides [6]. HUVECs were processed in 6 well plates and the treating process with Cap (1 μM), NE (0.5 μM) or designed concentrations of ACEi peptides (100–400 μM) for 24 h, or treated with both NE (0.5 μM) and 200 μM ACEi peptides for 24 h. NO and ET-1 production in treated cells were measured by employing human NO and ET-1 assay kit as manufactures’ protocol, separately.

### 3.8. Statistical Analysis

All data are expressed as the mean±standard deviation (SD) with triplicate and analyzed by SPSS 20.0 (SPSS Corporation, Chicago, IL, USA). Significant differences were obtained by employing ANOVA test with Dunnett or Tukey Test (*p*  <  0.05, *p*  <  0.01, or *p*  <  0.001).

## 4. Conclusions

In conclusion, the conditions of Alcalase for hydrolyzing the skipjack tuna muscle protein were optimized using single-factor and response-surface experiments, and six ACEi peptides were purified from the protein hydrolysate prepared under optimum conditions of Alcalase and were identified as SP, VDRYF, VHGVV, YE, FEM, and FWRV, respectively. SP and VDRYF displayed noticeable ACEi activity, attributed to the effective interaction with the active sites/pockets of ACE by hydrogen bonding, electrostatic force, and hydrophobic interaction. Moreover, SP and VDRYF could alleviate the negative effects of NE-constrained NO production and NE-induced ET-1 secretion. The mentioned results suggest the huge potential of ACEi peptides of skipjack tuna muscle for nutraceutical or therapeutic-related use to regulate cardiovascular disease. The findings also demonstrate one conductive influence exerted by marine natural proteins as potential ACEi peptide sources for antihypertensive treatment. Our further studies will explore the in vivo experiment of the prepared ACEi peptides for clarifying their mechanisms to control high blood pressure.

## Figures and Tables

**Table 1:** Amino acid composition of skipjack tuna (*Katsuwonus pelamis*) muscle (g/100g).

Amino Acid	Content (g/100g)	
	Undefatted Muscle	Defatted Muscle
Asp	8.369	8.936
Thr	4.034	4.316
Ser	3.394	3.655
Glu	12.843	13.785
Pro	2.845	3.070
Gly	3.683	4.128
Ala	4.988	5.417
Cys	0.443	0.507
Val	4.540	4.835
Met	2.390	2.528
Ile	4.026	4.285
Leu	7.205	7.620
Tyr	2.970	3.234
Phe	3.952	3.944
Lys	7.851	8.189
His	3.128	3.427
Arg	5.023	5.369
WTAA	81.686	87.245
WEAA	33.998	35.717
WEAA/WTAA (100%)	41.62	40.94
WHEAA	8.15	8.80
WNEAA	39.54	42.73
WEAA/WNEAA (100%)	85.99	83.59

AA, amino acid; WTAA, weight of total amino acid; WEAA, weight of essential amino acid; WHEAA, weight of half essential amino acid; WNEAA, weight of non-essential amino acid.

**Table 2:** Box–Behnken design and experimental results of response surface methodology.

Run	Independent Variables ^a^			Dependent Variables ^b^
	*X*_1_ (pH)	*X*_2_ (temperature/°C)	*X*_3_ (enzyme dose/%)	*Y* (ACEi rate %)
1	9	55	1.5	66.01
2	9	60	2	67.19
3	9.5	60	2.5	67.56
4	10	55	2.5	67.56
5	10	55	1.5	53.06
6	9	55	2.5	66.00
7	10	60	2	61.95
8	9.5	55	2	68.00
9	10	50	0	56.32
10	9.5	50	1.5	55.86
11	9.5	55	2	70.58
12	9	50	2	63.93
13	9.5	55	2	66.82
14	9.5	50	2.5	66.11
15	9.5	55	2	68.39
16	9.5	55	2	67.90
17	9.5	60	1.5	67.31

^a^ Independent variables: *X*_1_, pH; *X*_2_, temperature; *X*_3_, enzyme dose; ^b^ dependent variables: *Y*, ACEi rate.

**Table 3:** ANOVA for response surface quadratic model: estimated regression model of relationship between dependent variables and independent variables.

Source	Sum of Squares	df	Mean Square	*F*-Value	*p*-Value	Significant
Model	407.86	9	45.32	24.24	0.0002	significant
*X*_1_-pH	73.45	1	73.45	39.29	0.0004	
*X*_2_-Temperature	59.35	1	59.35	31.75	0.0008	
*X*_3_-Enzyme dose	78.06	1	78.06	41.76	0.0003	
*X_1_X_2_*	1.4	1	1.4	0.75	0.4148	
*X_1_X_3_*	52.64	1	52.64	28.16	0.0011	
*X_2_X_3_*	25	1	25	13.37	0.0081	
*X*_1_^2^	60.02	1	60.02	32.11	0.0008	
*X*_2_^2^	31.22	1	31.22	16.7	0.0047	
*X*_3_^2^	15.41	1	15.41	8.24	0.024	
Residual	13.09	7	1.87			
Lack of Fit	4.27	3	1.42	0.65	0.6256	not significant
Pure Error	8.82	4	2.2			
Cor Total	420.95	16				

**Table 4:** Amino acid sequences, molecular weights (MWs), and ACEi activity (IC_50_ value) of six isolated peptides (TMAP1-TMAP6) from protein hydrolysates of skipjack tuna muscle (TMPH).

	Retention Time (min)	Amino Acid Sequence	Observed MW/ Theoretical MW (Da)	IC_50_ (mg/mL)
TMAP1	8.590	Ser-Pro (SP)	202.3/202.2	0.06 ± 0.01 ^a^
TMAP2	10.950	Val-Asp-Arg-Tyr-Phe (VDRYF)	698.9/698.8	0.28 ± 0.03 ^a^
TMAP3	14.032	Val-His-Gly-Val-Val (VHGVV)	509.7/509.6	0.90 ± 0.16 ^b^
TMAP4	17.139	Tyr-Glu (YE)	310.4/310.3	0.80 ± 0.03 ^b^
TMAP5	22.258	Phe-Glu-Met (FEM)	425.6/425.5	2.18 ± 0.20 ^c^
TMAP6	25.209	Phe-Trp-Arg-Val (FWRV)	606.8/606.7	0.76 ± 0.10 ^b^

^a–c^ Values with same letters indicate no significant difference (*p* > 0.05). IC_50_ (half maximal inhibitory concentration) is the concentration of inhibitor required for 50% inhibition of ACE.

## Data Availability

Data are contained within the article.

## Abbreviations

ACE: angiotensin-I-converting enzyme; ACEi, angiotensin-I-converting enzyme inhibitory; ET-1, endothelin-1; NO, nitric oxide; CVD, cardiovascular diseases; MW, molecular weight; Ang, angiotensin; Cap, captopril; NE, norepinephrine; eNOS, endothelial NO synthase; FAPGG,N-[3-(2-Furyl)acryloyl]-Phe-Gly-Gly; TMAP1, Ser-Pro (SP);TMAP2, Val-Asp-Arg-Tyr-Phe (VDRYF); TMAP3, Val-His-Gly-Val-Val (VHGVV); TMAP4, Tyr-Glu (YE); TMAP5, Phe-Glu-Met (FEM); TMAP6, Phe-Trp-Arg-Val (FWRV); AA, amino acid; WTAA, weight of total amino acid; WEAA, weight of essential amino acid; WHEAA, weight of half essential amino acid; WNEAA, weight of non-essential amino acid; TMPH, protein hydrolysate of tuna muscle prepared using Alcalase under the optimum hydrolysis conditions; GPC, gel permeation chromatography; RP-HPLC, Reversed-phase high performance liquid chromatography; HUVECs, Human umbilical vein endothelial cells; MTT, 3-(4,5-Dimethylthiazol-2yl)-2,5-diphenyltetrazolium bromide.
